# Editorial for the Special Issue “Microbial Diversity and Microbial Resistance”

**DOI:** 10.3390/biology14040415

**Published:** 2025-04-13

**Authors:** Chrysoula (Chrysa) Voidarou, Athina Tzora

**Affiliations:** Laboratory of Animal Health, Food Hygiene and Quality, Department of Agriculture, University of Ioannina, 47100 Arta, Greece; tzora@uoi.gr

In the beginning, it was diversity—or so evolutionary biology suggest when interpreting how and why antibiotic production began on Earth. According to this epic narrative, *Streptomyces* species emerged approximately 440 million years ago, around the same time when the first plants took root on land. The ability to produce substances capable of eliminating competing microorganisms conferred a significant advantage in colonizing plant rhizospheres, a strategy which proved profoundly successful [1]. Even today, nearly two-thirds of clinically used antibiotics are derived from *Streptomyces* [2].

Bacterial communities are remarkably diverse, which critically influences both the emergence and spread of antibiotic resistance. Bacteria readily exchange genetic material through conjugation, transformation, and transduction—processes that enabling them to acquire new genes, including those which confer resistance to antimicrobial agents. Environmental reservoirs such as soil, water, and the human microbiome serve as key repositories for antibiotic resistance genes (ARGs) [3]. Once bacteria harboring ARGs encounter susceptible populations, resistance can propagate swiftly via horizontal gene transfer or competitive interactions. The concept of bacterial biodiversity encompasses more than the coexistence of multiple species; it also involves genetic variation within species and the functional traits that enable bacteria to thrive in countless ecological niches [4]. This expansive genetic repertoire provides a wealth of potential mutations and adaptations, some of which may confer resistance to antibiotics. Consequently, bacterial biodiversity is a double-edged sword: it underpins microbial adaptability and ecological success yet simultaneously accelerates the proliferation of antibiotic resistance.

Moreover, the broader genetic narrative extends beyond the direct interaction between antibiotics and bacteria. Diverse germicidal substances and various resistance elements—some autonomous, others embedded in chromosomes—constitute this microbial arsenal. As Toleman and Walsh (2011) aptly noted, the resistance determinants identified in pathogenic microorganisms serve as a “genetic document of the history of humankind’s chemical intervention with infectious disease.” [5]. Clinicians and public health experts caution that we may be on the cusp of returning to the pre-antibiotic era; research indicates that more than 20,000 potential resistance genes of nearly 400 different types have already been identified in sequenced bacterial genomes [6].

Bacterial biodiversity is thus integral to maintaining ecological balance and shapes the trajectory of antibiotic resistance. Within complex microbial communities, the abundance of genetic variants enables myriad resistance mechanisms to emerge, persist, and spread. Studies have shown that ARGs frequently move between bacteria via mobile genetic elements, especially plasmids, which play a crucial role in disseminating resistance traits across species boundaries [7]. This horizontal gene transfer is particularly prevalent in environments where antibiotics are heavily employed, such as agriculture and healthcare [3,8]. High microbial diversity can, in some cases, buffer the dominance of resistant strains by fostering competition [9]. Conversely, ecosystems experiencing biodiversity loss—often due to anthropogenic pressures—can become hotspots for the unchecked proliferation of antibiotic-resistant bacteria [10]. The introduction of antibiotics into these environments through agricultural runoff or wastewater discharge intensifies selection pressure, ultimately favoring resistant populations and reducing overall microbial diversity [10,11].

Building on groundbreaking discoveries which link microbial diversity with rising antibiotic resistance, the articles in this Special Issue offer a compelling, far-reaching perspective on a global crisis. From pristine ecological niches to high-stakes clinical settings, they reveal how resistance mechanisms, horizontal gene transfer, and policy innovations demand cohesive, interdisciplinary action. Taken together, these studies highlight the urgent need to safeguard public health and preserve the delicate balance of microbial ecosystems in our rapidly changing world.

Microbial diversity in environmental and ecological contexts encompasses a wide variety of habitats, each with pressing concerns related to microbial ecology and resistance. González-Reguero et al. (2023) demonstrate how plant-growth-promoting bacteria (PGPB) can help *Lupinus albus* adapt to mercury-contaminated soils while concurrently lowering antibiotic resistance levels in the surrounding community, thus opening new avenues for bioremediation and soil health [12]. Moving to tropical ecosystems, Nimnoi and Pongsilp (2022) employ next-generation sequencing to show how soil organic matter and salinity shape microbial diversity, offering crucial data to sustain mangrove forests facing increasing environmental pressures [13]. In a different setting, Loiko et al. (2022) document how chemical toilet additives have led to the emergence of highly biocide-resistant bacterial strains in fecal sludge, prompting the development of biologically based products to detoxify disinfectant-contaminated wastewater prior to conventional treatment [14]. Further inland, the study by Dong et al. (2022) documents the biodiversity and geographic distribution of plant-parasitic nematodes in turfgrass soil and clarifies the ecological factors driving their distribution, paving the way for sustainable control measures which could save substantial resources in the turf industry while preserving environmental balance [15]. Contrasting warmer climates, a study on Antarctic meltwater ponds (MPs) reveals that psychrotolerant, enzyme-producing bacteria not only thrive in alkaline, high-salinity conditions but also exhibit diverse antibiotic resistance profiles—from multi-resistant to fully susceptible strains—highlighting their potential as bioindicators for tracking antibiotic resistance gene mobilization and the impact of human or animal activity in polar regions [16].

Turning to yeast-based solutions, Hicks et al. (2021) showed that *Metschnikowia pulcherrima* exhibits broad-spectrum antimicrobial activity against bird-associated pathogens via multiple inhibitory mechanisms beyond pulcherrimin. Its secretome can modulate bacterial growth, revealing complex yeast–bacteria interactions and indicating promising biocontrol potential for agriculture and feed applications [17]. Salamandane et al. (2021) identified widespread fecal contamination and multidrug-resistant bacteria in Maputo’s drinking water, highlighting the risk of convergence between microbial diversity and antibiotic resistance in many Mozambican cities [18]. Finally, the study entitled “Genome Analysis of *Acinetobacter lwoffii* Strains Isolated from Permafrost Soils Aged from 15 Thousand to 1.8 Million Years” unveils that ancient Acinetobacter isolates share striking genomic similarities with modern environmental and clinical strains, exposing extensive horizontal gene transfer over millennia, underscoring the enduring, dynamic evolution of microbial life [19].

Moving into the critical intersection of microbial communities with agriculture and food systems, the study by Mohamed et al. (2022) underscores the threat posed by *A. baumannii* harboring notable β-lactam resistance, highlighting the urgent need for the systematic surveillance of dairy products [20]. Similarly, the study by Ammar et al. (2022), which sheds light on the prevalence and antimicrobial susceptibility of bovine *Mycoplasma* species in Egypt, reveals rising resistance to key antibiotics, emphasizing the need for more judicious veterinary antibiotic use [21]. Further heightening concerns about dairy safety, the study on *Mammaliicoccus* sp. from German dairy farms reveals that these often overlooked staphylococci-like bacteria harbor a broad array of AMR genes and exhibit non-wildtype phenotypes, posing a hidden threat to farm biosecurity through potential horizontal gene transfer to more pathogenic species of the family Staphylococcaceae, such as *S. aureus* [22].

Shifting the focus to small ruminants, the study “Isolation of *Listeria ivanovii* from Bulk-Tank Milk of Sheep and Goat Farms” indicates that isolates’ limited evolutionary diversity may translate into reduced pathogenicity, emphasizing the value of genomic monitoring for public health protection [23]. Complementing this, Lianou et al. (2021) reveal that resistant staphylococci in sheep milk can transmit cell-free resistance genes through dairy products—surviving thermal processing and transferring to human microbiota [24]. Another study documents a higher prevalence of coagulase-positive staphylococci (CoNS) over *S. aureus* in goat mastitis, along with significant antibiotic resistance profiles, underscoring serious implications for animal welfare and the microbiological safety of dairy products [25]. Further, using multilocus sequence typing analysis (MLST), a study reveals significant genetic diversity among *Staphylococcus epidermidis* strains causing ovine mastitis, alongside alarming levels of antimicrobial resistance. This variability highlights the inherent complexity of staphylococcal infections in small ruminants and underscores the need for advanced diagnostics and targeted interventions [26]. Beyond mammalian livestock, the study “Serological Variety and Antimicrobial Resistance in *Salmonella* Isolated from Reptiles” demonstrates that both wild and domesticated reptiles can harbor multidrug-resistant *Salmonella* serovars [27]. Building on these findings, Bendary et al. (2022) shift their focus to *Clostridium perfringens* by examining isolates from meat, poultry, and dairy products and analyzing their toxin-genotype diversity. They reveal a high prevalence of multidrug-resistant strains, underscoring the urgent need for further research to solidify correlations between resistance and toxinotypes [28]. Another study reveals that *Proteus mirabilis* from foxes, raccoons, and minks harbors carbapenemase genes (*bla*_OXA-24_ in 15.09% and *blaNDM* in 13.21%), highlighting fur animals as reservoirs of resistance and emphasizing the need for wildlife monitoring in One Health frameworks [29]. Finally, in seafood, Iacumin et al. (2022) demonstrate that combining vacuum or modified-atmosphere packaging with *Latilactobacillus sakei* significantly enhances the microbiological quality of gutted sea bass and sea bream, showcasing the synergy of mild preservation methods in safeguarding seafood [30].

Within the Clinical and One Health Perspectives section, several studies illuminate the complex interplay of microbial adaptation and resistance across human and animal hosts. The study by Bendary et al. (2022) sheds light on the daunting challenge of managing diarrheagenic *Ecerichia coli* (DEC) pathotypes across human and animal hosts [31]. They reveal a strong correlation between DEC pathotypes, virulence markers, and multidrug resistance, pinpointing these factors as key determinants of morbidity and essential guides for treatment. Research on the brain natriuretic peptide (BNP) reveals dose-dependent enhancements in bacterial resilience, affecting growth, survival, and antibiotic resistance, thereby holding promise for clinical applications and the development of robust bacterial preparations in agriculture, medical practice, and waste management [32]. Meanwhile, the investigation of non-*faecalis*, non-*faecium Enterococcus* species (including *E. raffinosus*, *E. durans*, and *E. avium*) from a Romanian tertiary care hospital reveals an alarming emergence of antibiotic-resistant enterococci. This finding underscores their escalating role as nosocomial pathogens and urgently calls for the refinement of diagnostic protocols [33]. In the COVID-19 era, profiling bacterial infections reveal a surge in secondary infections marked by heightened antimicrobial resistance, notably in *Klebsiella pneumoniae* and *Acinetobacter* spp, underscoring the urgent need for targeted antimicrobial stewardship during the pandemic [34]. Additionally, studies on *Klebsiella pneumoniae* reveal that high-risk, colistin-resistant clones possess iron uptake systems that facilitate neutrophil evasion, suggesting that targeting these pathways may offer a promising treatment for hypervirulent infections [35]. Meanwhile, ESBL-producing strains from hospitalized patients, poultry farms, and farm workers in Egypt exhibit alarming co-resistance traits, demanding coordinated interventions across veterinary and medical sectors [36].

Finally, two comprehensive reviews expand our understanding of multidrug resistance and microbial balance. Borgio et al. (2021) systematically review the incidence of MDR bacteria and fungi across nine Arabian Peninsula countries, emphasizing region-specific diagnostics and surveillance in human, animal, agricultural, and environmental domains [37]. Ersanli et al. (2023) highlight the skin microbiota as a vital biomarker for wound healing, underscoring the essential role of microbial balance in tissue repair. Together, these reviews bridge clinical, ecological, and agricultural perspectives, paving the way for integrated strategies to mitigate multidrug-resistant organism (MDRO) threats and enhance overall well-being [38].

These articles collectively illustrate the breadth of contemporary research on microbial diversity and antimicrobial resistance—ranging from remote Antarctic ponds to hospital intensive care units. They underscore the interconnectedness of environmental, agricultural, and clinical contexts, echoing the critical need for multidisciplinary and holistic strategies to manage and mitigate microbial resistance around the globe.
